# Clinical characteristics and initial management of patients with tuberculous pericarditis in the HIV era: the Investigation of the Management of Pericarditis in Africa (IMPI Africa) registry

**DOI:** 10.1186/1471-2334-6-2

**Published:** 2006-01-06

**Authors:** Bongani M Mayosi, Charles Shey Wiysonge, Mpiko Ntsekhe, Jimmy A Volmink, Freedom Gumedze, Gary Maartens, Akinyemi Aje, Baby M Thomas, Kandathil M Thomas, Abolade A Awotedu, Bongani Thembela, Phindile Mntla, Frans Maritz, Kathleen Ngu Blackett, Duquesne C Nkouonlack, Vanessa C Burch, Kevin Rebe, Andy Parish, Karen Sliwa, Brian Z Vezi, Nowshad Alam, Basil G Brown, Trevor Gould, Tim Visser, Muki S Shey, Nombulelo P Magula, Patrick J Commerford

**Affiliations:** 1 The Cardiac Clinic, Department of Medicine, University of Cape Town, E25 Groote Schuur Hospital, Observatory 7925, South Africa; 2 Primary Health Care Directorate, University of Cape Town, Cape Town, South Africa; 3 Department of Statistical Sciences, University of Cape Town, Cape Town, South Africa; 4 Division of Pharmacology, Department of Medicine, University of Cape Town, Cape Town, South Africa; 5 Department of Cardiology, University College Hospital, Ibadan, Nigeria; 6 Department of Medicine, Nelson Mandela Academic Hospital and Walter Sisulu University, Mthatha, South Africa; 7 Department of Medicine, Prince Mshiyeni Hospital, Durban, South Africa; 8 Department of Cardiology, MEDUNSA, Pretoria, South Africa; 9 Department of Internal Medicine, Karl Bremer Hospital, Bellville, South Africa; 10 Faculty of Medicine and Biomedical Sciences, University of Yaoundé I and Centre Hospitalier et Universitaire, Yaoundé, Cameroon; 11 Department of Medicine, GF Jooste Hospital, Cape Town, South Africa; 12 Cecilia Makiwane Hospital, East London, South Africa; 13 Department of Cardiology, Chris Hani Baragwanath Hospital and University of the Witwatersrand, Soweto, South Africa; 14 Subdepartment of Cardiology, Inkosi Albert Luthuli Central Hospital and University of KwaZulu Natal, Durban, South Africa; 15 Livingstone's Hospital, Port Elizabeth, South Africa; 16 Provincial Hospital, Port Elizabeth, South Africa; 17 Department of Medicine, George Hospital, George, South Africa; 18 Eersterivier Hospital, Cape Town, South Africa; 19 Mycobacterial Immunology Group, Institute of Infectious Diseases and Molecular Medicine, University of Cape Town, Cape Town, South Africa; 20 Subdepartment of Infectious Diseases, Department of Medicine, King Edward VIII Hospital and University of KwaZulu Natal, Durban, South Africa

## Abstract

**Background:**

The incidence of tuberculous pericarditis has increased in Africa as a result of the human immunodeficiency virus (HIV) epidemic. However, the effect of HIV co-infection on clinical features and prognosis in tuberculous pericarditis is not well characterised. We have used baseline data of the Investigation of the Management of Pericarditis in Africa (IMPI Africa) registry to assess the impact of HIV co-infection on clinical presentation, diagnostic evaluation, and treatment of patients with suspected tuberculous pericarditis in sub-Saharan Africa.

**Methods:**

Consecutive adult patients in 15 hospitals in three countries in sub-Saharan Africa were recruited on commencement of treatment for tuberculous pericarditis, following informed consent. We recorded demographic, clinical, diagnostic and therapeutic information at baseline, and have used the chi-square test and analysis of variance to assess probabilities of significant differences (in these variables) between groups defined by HIV status.

**Results:**

A total of 185 patients were enrolled from 01 March 2004 to 31 October 2004, 147 (79.5%) of whom had effusive, 28 (15.1%) effusive-constrictive, and 10 (5.4%) constrictive or acute dry pericarditis. Seventy-four (40%) had clinical features of HIV infection. Patients with clinical HIV disease were more likely to present with dyspnoea (odds ratio [OR] 3.2, 95% confidence interval [CI] 1.4 to 7.4, P = 0.005) and electrocardiographic features of myopericarditis (OR 2.8, 95% CI 1.1 to 6.9, P = 0.03). In addition to electrocardiographic features of myopericarditis, a positive HIV serological status was associated with greater cardiomegaly (OR 3.89, 95% CI 1.34 to 11.32, P = 0.01) and haemodynamic instability (OR 9.68, 95% CI 2.09 to 44.80, P = 0.0008). However, stage of pericardial disease at diagnosis and use of diagnostic tests were not related to clinical HIV status. Similar results were obtained for serological HIV status. Most patients were treated on clinical grounds, with microbiological evidence of tuberculosis obtained in only 13 (7.0%) patients. Adjunctive corticosteroids were used in 109 (58.9%) patients, with patients having clinical HIV disease less likely to be put on them (OR 0.37, 95% CI 0.20 to 0.68). Seven patients were on antiretroviral drugs.

**Conclusion:**

Patients with suspected tuberculous pericarditis and HIV infection in Africa have greater evidence of myopericarditis, dyspnoea, and haemodynamic instability. These findings, if confirmed in other studies, may suggest more intensive management of the cardiac disease is warranted in patients with HIV-associated pericardial disease.

## Background

Pericarditis is a common disorder that has multiple causes and presents in various clinical settings [1]. Tuberculosis is responsible for more than 50% of cases of pericarditis in developing countries where tuberculosis remains a major public health problem [2]. By contrast, *Mycobacterium tuberculosis *infection accounts for less than 5% of cases of pericarditis in industrialised countries [1]. In Africa, the incidence of tuberculous pericarditis is said to be rising as a direct result of the human immunodeficiency virus (HIV) epidemic [3-7]. There is a strong association between HIV infection and tuberculous pericarditis in endemic regions, where 40–75% of patients with large pericardial effusion (suspected to be tuberculosis) are infected with HIV [2,3].

The effect of HIV infection on the clinical presentation, response to treatment and outcome of patients with tuberculous pericarditis is not well characterised [8]. HIV infected patients with tuberculous pericarditis have been found to be more likely than HIV negative patients to have disseminated tuberculosis, raising the possibility that dissemination may worsen long-term outcome [5]. Preliminary evidence suggests that HIV infection may be associated with higher mortality in tuberculous pericarditis; mortality with anti-tuberculosis chemotherapy ranged from 8% to 17% in the pre-HIV era [9-12], whereas higher mortality rates of 17–34% have been reported in HIV infected individuals [13].

We have conducted the first African multi-centre prospective observational registry of the clinical presentation, diagnostic evaluation, initial treatment, and outcome of patients with suspected tuberculous pericarditis in the HIV era. This study, which is called the *I*nvestigation of the *M*anagement of *P*ericarditis *i*n *Africa *(IMPI Africa) Registry, was established mainly to obtain contemporary information that is required for the design of a clinical trial of the effectiveness of adjunctive steroids in tuberculous pericarditis in the HIV era [2]. In this report we have used the baseline data of the IMPI Africa Registry to assess the impact of clinical HIV disease on the clinical characteristics, diagnostic work-up, and initial treatment of patients with suspected tuberculous pericarditis.

## Methods

The IMPI Africa registry is a simple pragmatic multi-centre prospective observational study of patients admitted to hospital with suspected tuberculous pericarditis which was designed to assess the impact of HIV infection on clinical presentation, diagnostic evaluation, initial treatment, and outcome of patients with tuberculous pericarditis in Africa. We set out to enrol a minimum of 100 consecutive suspected tuberculous pericarditis patients presenting to collaborating physicians over 6 months, and follow them prospectively for 6–12 months. The study was approved by the research ethics committee of the University of Cape Town, South Africa, and all participants gave written informed consent.

Twenty seven hospital-based physicians from eight African countries (Cameroon, 2; Ghana, 1; Kenya, 1; Lesotho, 1; Nigeria, 1; South Africa, 19; Uganda, 1; Zimbabwe, 1) were invited by electronic mail in January 2004 to participate in the study. These physicians had expressed an interest in the project to one of the investigators (BMM). Fifteen physicians from three countries (Cameroon, 1; Nigeria, 1; and South Africa, 13) contributed patients to the IMPI Africa Registry (56% response rate). Twelve of the 15 physicians were affiliated to medical schools, and three (all in South Africa) were based in district general hospitals.

Suspected tuberculous pericarditis was defined as a patient presenting to hospital with a clinical syndrome of pericardial disease which was suspected to be caused by tuberculosis on the basis of clinical and/or laboratory findings leading to the commencement of anti-tuberculosis chemotherapy according to the national tuberculosis control programme [14] Consecutive incident cases of suspected tuberculous pericarditis were enrolled. Adult patients were eligible for inclusion in the study if the collaborating physician felt sufficiently confident with the diagnosis of tuberculous pericarditis to commence anti-tuberculosis treatment. The management of each patient was at the discretion of the collaborating physician in keeping with the observational nature of the study.

Pericardial disease was classified as acute non-effusive pericarditis, pericardial effusion, effusive-constrictive pericarditis, or constrictive pericarditis on the basis of the collaborating physician's assessment of clinical and imaging information.

We classified tuberculous pericarditis as 'definite' or 'probable' depending on the amount of clinical and bacteriological information supporting the diagnosis of tuberculosis, or 'not tuberculous pericarditis' when an alternative (non-tuberculous) cause of pericarditis was found. A diagnosis of 'definite' tuberculous pericarditis was based on the demonstration of tubercle bacilli in pericardial fluid (smear and/or culture) or on histologic section of the pericardium; 'probable' tuberculous pericarditis on the proof of tuberculosis elsewhere in a patient with otherwise unexplained pericarditis, on the basis of indirect tests (e.g., adenosine deaminase level ≥ 40 IU/l in pericardial fluid) and/or an appropriate response to a trial of anti-tuberculosis chemotherapy.

The HIV status of the patients was based on the results of serological testing for HIV. However, serological testing for HIV is not always available or offered in some medical institutions in Africa. Therefore, the physicians were requested to state whether they suspected HIV infection on clinical grounds and to classify each patient as either having evidence of 'clinical HIV disease' or 'no clinical HIV disease' without regard to the HIV serological status of the patient. This assessment was left to the discretion of the collaborating physician, and no criteria were specified.

The effect of pericardial disease on the functional capacity of each patient was defined using New York Heart Association (NYHA) criteria as class I (no limitation in physical activity), class II (ordinary physical activity results in dyspnoea), class III (minimal physical activity results in dyspnoea), or class IV (dyspnoea at rest) [15]. Haemodynamic instability was defined by the presence of at least two of these signs: pulse rate > 100 beats/minute, systolic blood pressure < 100 mmHg, and cardiac tamponade requiring pericardiocentesis. The radiological, electrocardiographic, echocardiographic, and laboratory results were based on the report provided by the collaborating physician without central verification.

Demographic, clinical, diagnostic and therapeutic information was captured by means of a standardized data collection form (available on request) and transmitted (by fax or e-mail) to the IMPI Africa Coordinating Centre at the Cardiac Clinic, Groote Schuur Hospital, Cape Town, South Africa. Enrolment into the registry started on 1 March 2004 in 11 hospitals and up to two months later in the other four, and ended on 31 October 2004. Patients were reviewed for the study outcomes at three, six and 12 months following entry into the registry, with a minimum follow-up period of 6 months for all participants. Patient follow-up was carried out by personal interview, failing which telephonic enquiry and postal services were used.

On enrolment, each patient provided a contact person (next of kin or neighbour) who could be contacted if the patient's whereabouts were not known at the time of follow-up. In the event of death or illness the contact person would be able to provide information. In the event of death, every effort was made to obtain a copy of the death certificate. We also used the services of the South African Department of Home Affairs (for South African centres) and a private detective company to obtain the vital status of patients lost to follow-up. The follow-up phase of the study ended on 30 April 2005, and (follow-up) data will be reported separately.

Data were analysed using Epi Info 3.3 (CDC, Atlanta, GA, USA). We used the chi-square test (or Fisher's exact test for variables with small number of expected frequencies) and analysis of variance to asses differences between categorical and continuous variables respectively. Significance tests were two-tailed and statistical significance was defined at the 5% alpha level. All patients were stratified by clinical HIV disease status and entered in the analyses regardless of the final diagnosis.

## Results

### Clinical profile

One hundred and eighty-five patients were recruited, with the number recruited per geographic region varying from 17 (9.2%) in Yaoundé, Cameroon to 58 (31.4%) in the Eastern Cape Province of South Africa (Table 1). The general characteristics of the study population are shown in Table 2, stratified by clinical HIV disease status. The median age of the patients at diagnosis was 33 (range 14–87) years, and 82 (56%) were men. The proportions of patients in NYHA functional classes I to IV were 21.1% (39/185), 37.3% (69/185), 25.4% (47/185), and 16.2% (30/185), respectively. Pericardial effusion was diagnosed in 79.5%, effusive-constrictive pericarditis in 15.1%, acute dry pericarditis in 3.8%, and constrictive pericarditis in 1.6%. Haemodynamic instability was reported in 49 (26.5%) patients, 38 (77.6%) of whom required pericardiocentesis to relieve cardiac tamponade.

**Table 1:** Geographic distribution of the hospitals and study population

**Country**	**Region**	**Hospital**	**Number of patients recruited (%)**
Cameroon	Centre	Centre Hospitalier et Universitaire, Yaoundé	17 (9.2)
Nigeria	South West	University College Hospital, Ibadan	31 (16.8)
South Africa	Eastern Cape	Nelson Mandela Academic, Umtata	42 (22.7)
		Livingstone and Provincial, Port Elizabeth	10 (5.4)
		Cecilia Makiwane, East London	6 (3.2)
	Western Cape	Groote Schuur and GF Jooste, Cape Town	17 (9.2)
		Karl Bremer, Bellville	13 (7.0)
		George, George	3 (1.6)
		Eersterivier, Cape Town	2 (1.1)
	Guateng	Dr George Mukhari, Tshwane	15 (8.1)
		Chris Hani Baragwanath, Johannesburg	6 (3.2)
	KwaZulu Natal	Prince Mshiyeni, Durban	16 (8.6)
		King Edward VIII, Durban	7 (3.8)

Total			185 (100.0)

**Table 2:** Clinical characteristics of the study population by clinical HIV status

	**Clinical HIV disease**	**No clinical HIV disease**	**P**
Number of patients	74 (40)	111 (60)	
Age (median, range), years	36 (18–87)	32 (15–79)	0.14
Gender			
Men	42 (40.8)	61 (59.2)	
Women	32 (39.0)	50 (61.0)	0.81
Region			
Eastern Cape, SA^+^	20 (34.5)	38 (65.5)	
Western Cape, SA	11 (31.4)	24 (68.6)	
Ibadan, Nigeria	6 (19.4)	25 (80.6)	0.0001
KwaZulu Natal, SA	11 (47.8)	12 (52.2)	
Gauteng, SA	11 (52.4)	10 (47.6)	
Yaoundé, Cameroon	15 (88.2)	2 (11.8)	
Pericardial syndrome			
Acute	1 (14.3)	6 (85.7)	
Effusion	65 (44.2)	82 (55.8)	
Effusive-constrictive	8 (28.6)	20 (71.4)	
Constrictive	0 (0.0)	3 (100.0)	0.09
NYHA^++ ^Functional Class			
I	8 (20.5)	31 (79.5)	
II	27 (39.1)	42 (60.9)	
III	20 (42.6)	26 (57.4)	
IV	19 (63.3)	11 (36.7)	0.004
Haemodynamic instability**			
Yes	24 (49.0)	25 (51.0)	
No	50 (36.8)	86 (63.2)	0.13
Tamponade requiring centesis	17 (44.7)	21 (55.2)	0.97

Values are median (range) and absolute counts (percentages)

^+^SA, South Africa; ^++^NYHA, New York Heart Association (I, No limitation of physical activity; II, Slight limitation of physical activity; III, Marked limitation of physical activity; and IV, Unable to carry out any physical activity without discomfort); **Pulse rate more than 100 bpm, Systolic blood pressure less than 100 mmHg and or tamponade requiring centesis.

Seventy-four (40.0%) patients had clinical evidence of HIV disease. There was significant geographical variation in the proportion of patients with clinical HIV disease (P = 0.0001), with Cameroon having the highest (88.2%) and Nigeria the lowest (19.4%). In South Africa, patients recruited from the Gauteng Province had the highest prevalence of clinical HIV disease (52.4%), and the Western Cape Province the lowest (31.4%). Patients with clinical HIV disease were more likely to present in worse dyspnoea (P = 0.004). There was no significant association between age, gender, haemodynamic stability and clinical HIV disease (Table 2).

We evaluated the ability of the physicians to predict serological HIV status by using clinical criteria of HIV disease in a subset of 96 patients who were tested for HIV. There was strong agreement between clinical signs of HIV disease and serological HIV status (Table 3). The sensitivity of clinical signs of HIV disease was 75.5%, specificity 88.4%, positive predictive value 88.9%, and negative predictive value 74.5%, which compare favourably with previous studies in Africa [16]. There was, however, a bias in HIV testing, with tests done in 53/74 (72%) of cases with clinical HIV disease and 43/111 (39%) of cases without clinical HIV disease (p < 0.001). This bias may have affected the findings for sensitivity and specificity of clinical diagnosis and may have influenced the reliability of observations regarding associations with HIV serostatus.

**Table 3:** Ability of collaborating physicians to predict HIV serological status from clinical assessment of HIV disease

	**HIV Serological Status**		
**Clinical HIV Disease**	Positive	Negative	

Yes	40	5	**45**
No	13	38	**51**
	**53**	**43**	**96**

Sensitivity: 40/53 = 75.5%

Specificity: 38/43 = 88.4%

Positive predictive value: 40/45 = 88.9%

Negative predictive value: 38/51 = 74.5%

### Diagnostic evaluation

The chest radiograph and echocardiogram were performed in 179 (96.8%) and 174 (94.1%) patients, respectively. Electrocardiography (ECG) was carried out in 119 (64.3%) patients, and only 69 (37.3%) were subjected to pericardiocentesis. There was no significant variation in the diagnostic tests used by clinical HIV disease status (Table 4, 5, 6). Forty-eight (26.8%) patients had radiological signs of active pulmonary tuberculosis (Table 4). Patients with clinical HIV disease were more likely to have radiological signs of active pulmonary tuberculosis (P = 0.003). The most prevalent ECG changes were micro voltage (22.7%), S-T segment elevation (20.2%), and P-R segment deviation (16.8%). Atrial fibrillation and electrical alternans were present in 12 (10.1%) and 11 (9.2%) patients with ECGs, respectively (Table 5). There was no statistically significant relationship between clinical HIV disease and atrial fibrillation (P = 0.09), electrical alternans (P = 0.43), micro voltage (P = 0.27), and PR segment deviation (P = 0.09). However, clinically HIV infected patients were more likely to have ST segment elevation (P = 0.03), which is indicative of myocardial injury.

**Table 4:** Chest X-ray changes in the study population by clinical HIV status

**Feature**	**Clinical HIV disease**	**No clinical HIV disease**	**P**
Number of patients Cardiomegaly	72 (40.2)	107 (59.8)	0.39
Yes	62 (42.2)	85 (57.8)	0.25
No	10 (31.3)	22 (68.8)	
Pericardial calcification			
Yes	1 (20.0)	4 (80.0)	0.33
No	71 (40.8)	103 (59.2)	
Active PTB*			
Yes	28 (58.3)	20 (41.7)	0.003
No	44 (33.6)	87 (66.4)	

Values are absolute counts (percentages)

* PTB: Pulmonary tuberculosis

**Table 5:** Electrocardiographic changes in study population by clinical HIV status

**Feature**	**Clinical HIV disease**	**No clinical HIV disease**	**P**
Number of patients PR segment deviation	46 (38.7)	73 (61.3)	0.62
Yes	11 (55.0)	9 (45.0)	0.09
No	35 (35.4)	64 (64.6)	
ST segment elevation			
Yes	14 (58.3)	10 (41.7)	0.03
No	32 (33.7)	63 (66.3)	
Micro voltage			
Yes	8 (29.6)	19 (70.4)	0.27
No	38 (41.3)	54 (58.7)	
Electrical alternans			
Yes	5 (45.5)	6 (54.5)	0.43
No	41 (38)	67 (62)	
Atrial fibrillation			
Yes	2 (16.7)	10 (83.3)	0.09
No	44 (41.1)	63 (58.9)	

Values are absolute counts (percentages)

**Table 6:** Results of pericardial fluid analyses by clinical HIV status

**Feature**	**Clinical HIV disease**	**No clinical HIV disease**	**P**
Pericardiocentesis Indication:	31 (44.9)	38 (55.1)	0.29
Diagnostic	14 (45.2)	17 (54.8)	0.97
Therapeutic	17 (44.7)	21 (55.3)	
Pericardial aspirate analyses:			
Adenosine deaminase (n)			
> 40 IU/L	8 (42.1)	11 (57.9)	0.79
< 40 IU/L	7 (46.7)	8 (53.3)	
Ziehl-Neelsen stain for acid-fast bacilli (n)			
Positive	3 (42.9)	4 (57.1)	0.62
Negative	20 (40.8)	29 (59.2)	
TB culture (n)			
Positive	2 (33.3)	4 (66.7)	0.61
Negative	4 (40.0)	6 (60.0)	

Values are absolute counts (percentages)

IU/L: international units per litre.

Sixty nine patients had pericardiocentesis for diagnostic (N = 31) or therapeutic (N = 38) reasons (Table 6). Results of chemistry (adenosine deaminase [ADA]) and microscopic analyses (Ziehl-Neelsen stain for acid fast bacilli), and *Mycobacterium tuberculosis *culture were available for 35, 55, and 16 patients, respectively. Thirteen (7.0%) patients had definite tuberculous pericarditis, 166 (89.7%) probable tuberculous pericarditis, and six (3.2%) patients had an alternative diagnosis. There were no significant differences between clinically HIV infected patients and those who were not with regard to having a high ADA level (> 40 IU/L) or presence of acid fast bacilli on microscopic analysis (P = 0.79 and P = 0.61, respectively).

### Initial treatment

One hundred and seventy-eight patients (96.2%) were on four-drug anti-tuberculosis chemotherapy for new tuberculosis cases and the rest were on five drugs for re-treatment of tuberculosis. A total of 109 (58.9%) patients were put on adjunctive corticosteroids. Clinically HIV infected patients were less likely to be put on an adjunctive corticosteroid (P = 0.001). Seven patients (10% of patients with clinical HIV disease) received antiretroviral drugs, all of whom were recruited in the non-South African centres. All drugs (anti-tuberculosis, corticosteroids, and antiretroviral drugs) were given orally. In particular, none of the patients who received steroids were administered these agents by intra-pericardial or intravenous routes.

### Findings in the sub-group with known HIV serological status

HIV serological tests were conducted in 96 participants (55%); there were no significant age and sex differences between participants with and without HIV serological results. There were, however, major regional differences in the proportions of participants who were tested for HIV, with Nigeria and Cameroon having the highest testing rates of 94% of participants tested in each centre, 66% in the Western Cape, 29% respectively in Gauteng and the Eastern Cape, and 22% in Kwa Zulu Natal. Results of the analyses by HIV serological status are shown in additional file 1 (Tables S1-S4). Like patients with clinical evidence of HIV infection, those who had a serological diagnosis of HIV were more likely to have dyspnoea (P = 0.02) and ST segment elevation on the electrocardiogram (P = 0.01). In addition, patients with serological evidence of HIV infection were more likely to have haemodynamic instability (P = 0.0008) and radiological signs of cardiomegaly (P = 0.01) than HIV uninfected patients, but there was no relationship between active pulmonary tuberculosis and serological HIV status (P = 0.19).

## Discussion

This prospective observational study indicates that 40% of patients presenting to hospital with suspected tuberculous pericarditis in parts of Cameroon, Nigeria and South Africa have clinical features of HIV infection. This study reveals for the first time that patients with clinical features of HIV infection have a different clinical presentation of the cardiac disease. Patients with clinical HIV disease and tuberculous pericarditis have greater dyspnoea and electrocardiographic ST segment changes that are suggestive of myopericarditis. In addition, a positive HIV serostatus was associated with greater cardiomegaly and haemodynamic instability in these patients. These data suggest that there may be greater pericardial fluid accumulation and myocardial involvement in patients with HIV-associated pericardial tuberculosis. These findings, if confirmed in prospective studies with less biased assessment of HIV status, suggest that tuberculous pericarditis is a more severe cardiac disease in people with HIV/AIDS.

Preliminary evidence from small single centre studies of tuberculous pericarditis in African patients infected with HIV has suggested that tuberculous pericarditis occurs in the early stages of HIV disease. In a Tanzanian study of HIV and tuberculous pericarditis, only 5 of the 28 HIV infected patients had clinical signs of HIV infection, suggesting that pericardial disease was an early manifestation of HIV infection in Tanzania [3]. Our finding that nearly half of the patients with suspected tuberculous pericarditis have overt features of HIV infection may be in keeping with the maturity of the HIV/AIDS epidemic in many parts of Africa, which is associated with a greater proportion of patients presenting in advanced stages of HIV disease than was the case 15 years ago when many of the studies were conducted [3-7]. There was, however, marked geographic variation in the prevalence of clinical HIV disease in our study which correlated with the known epidemiology of the HIV epidemic in Africa, with the exception of the Cameroon. In Cameroon we found a prevalence of clinical HIV disease in patients with suspected tuberculous pericarditis of 88.2%, compared to a much lower HIV seroprevalence of 5.5% in the general population [18].

There is a strong association between HIV infection and extra-pulmonary tuberculosis; about 67% of patients with extra-pulmonary tuberculosis are HIV infected in Zaire [17]. In Tanzania, 72% of patients with pericardial effusion were HIV seropositive, suggesting that pericardial effusion is strongly associated with HIV infection [3]. Of the 96 patients who were tested for HIV in the IMPI Africa registry, 53 (55.2%) were HIV infected.

A study of 88 patients from the Western Cape Province of South Africa, which included 39 HIV infected patients, suggested that there were no differences in the electrocardiographic findings between HIV infected and HIV negative patients with tuberculous pericarditis [19]. By contrast, we observed that more patients with clinical HIV disease than those without had ST segment elevation on ECG (30.4% *versus *13.7%), and there was a trend towards greater PR segment deviation, changes which are associated with involvement of the superficial layers of the myocardium in pericarditis [20]. The association of HIV infection with ST elevation on ECG was confirmed when the analyses were restricted to the sub-group of patients with known HIV sero-status.

In the pre-HIV era, changes of acute pericarditis in tuberculous pericarditis were reported in a small minority of patients (i.e., 9–11%) [19,21,22]. Whether the greater prevalence of electrical evidence of acute pericarditis bears relation to greater myocardial involvement in the form of a myopericarditis in immunocompromised patients remains to be established by prospective study [23-25]. It is possible that left ventricular dysfunction, associated with myopericarditis in patients with tuberculous pericarditis and HIV, may account for the greater dyspnoea that was observed in these patients.

### What are the limitations of the registry?

Our study methods can be criticised on several grounds. First, the economic circumstances of medical practice in many African countries result in frequent shortages that, in this study, preclude complete data acquisition in every patient. For example, 6% of patients enrolled in the registry did not have access to echocardiography for confirmation of the diagnosis of pericardial disease. Secondly, the lack of bacteriological confirmation of tuberculosis in the vast majority and HIV serology tests in half of the patients results from similar considerations. It should be noted, however, that the most intense investigation may fail to yield clear information as to aetiology in patients presenting with pericardial effusion [26]. The associations with clinical HIV disease should be interpreted with caution, and, given the low percentage tested, even associations with HIV seropositivity may have been subject to bias. This was a simple, large, prospective, observational study in a resource poor environment in which the definition of clinical and laboratory abnormalities were based on self-reports of the collaborating physicians without the verification of the primary data at a central site.

## Conclusion

### What are the implications for clinical practice?

Patients with suspected tuberculous pericarditis and HIV infection have evidence of a myopericarditis, poorer functional class, and haemodynamic instability in Africa. These factors are likely to be associated with a poorer prognosis, and may identify a high risk group that requires more intensive management of the cardiac disease and consideration of earlier treatment of the HIV disease.

### What are the implications for research?

There are at least three research questions that arise from this work. First, the impact of HIV infection on outcome in tuberculous pericarditis has not been addressed in long-term studies. The results of the follow-up of patients enrolled in this study should provide useful information in this regard. Second, the impression that tuberculous pericarditis is a myopericarditis in people with advanced HIV disease requires confirmation in prospective studies. Finally, the inconsistent use of adjunctive corticosteroids in patients with suspected tuberculous pericarditis illustrates the controversy surrounding the effectiveness of these agents in this setting [26]. A large randomised controlled trial of the effectiveness of adjunctive corticosteroids is required to address this issue, particularly in HIV infected individuals.

## Competing interests

The authors declare that they have no competing interests.

## Authors' contributions

The IMPI Africa Operations Committee (BMM [Chair], CSW [Coordinator], MN, JAV, GM, FG, PJC) designed the study, coordinated data collection, analyses and interpretation, and wrote the first draft of the manuscript. The IMPI Africa Steering Committee (BMM [Chair], CSW, MN, AA, BMT, BT, KNB, KR, KS, BGB) supervised all aspects of the study. All authors contributed intellectually to, read, and approved the final manuscript.

## Pre-publication history

The pre-publication history for this paper can be accessed here:



## Supplementary Material

Additional File 1 Findings in the sub-group with known HIV serological status. This file contains four tables (Table S1 to Table S4) which show the results of a subgroup analysis of the clinical characteristics and initial management of patients with tuberculous pericarditis. Click here for file
