# Supplementary material for: Clinical characteristics and initial management of patients with tuberculous pericarditis in the HIV era: the Investigation of the Management of Pericarditis in Africa (IMPI Africa) registry
Source: BMC Infect Dis. 2006 Jan 6;6:2. doi: 10.1186/1471-2334-6-2 (PMC1352368; doi:10.1186/1471-2334-6-2)
Supplement: Additional File 1 — Findings in the sub-group with known HIV serological status. This file contains four tables (Table S1 to Table S4) which show the results of a subgroup analysis of the clinical characteristics and initial management of patients with tuberculous pericarditis. [file 1471-2334-6-2-S1.doc]

# Findings in the sub-group with known HIV serological status

## Table S1: Clinical characteristics of the study population by serological HIV status

|  | **HIV positive** | **HIV negative** | **P** |
| --- | --- | --- | --- |
| Number of patients  Age (median, range), years  Gender   - Men - Women  Region  - Eastern Cape, SA^+^ - Western Cape, SA - Ibadan, Nigeria - KwaZulu Natal, SA - Gauteng, SA - Yaoundé, Cameroon  Pericardial syndrome  - Acute - Effusion - Effusive-constrictive - Constrictive   NYHA^++^ Functional Class   - I - II - III - IV   Haemodynamic instability**   - Yes - No   Tamponade requiring centesis | 53 (55.2)  33 (16-54)  32 (61.5)  21 (47.7)  15 (88.2)  14 (60.9)  3 (10.3)  4 (80.0)  3 (50.0)  14 (87.5)  1 (50.0)  46 (56.8)  6 (50.0)  0 (0.0)  8 (34.8)  24 (55.8)  10 (55.6)  11 (91.7)  17 (89.5)  36 (46.8)  10 (66.7) | 43 (44.8)  32 (15-70)  20 (38.5)  23 (52.3)  2 (11.8)  9 (39.1)  26 (89.7)  1 (20.0)  3 (50.0)  2 (12.5)  1 (50.0)  35 (43.2)  6 (50.0)  1 (100.0)  15 (65.2)  19 (44.2)  8 (44.4)  1 (8.3)  2 (10.5)  41 (53.2)  5 (33.3) | 0.32  0.18  <0.0001  0.69  0.02  0.0008  0.12 |

Values are median (range) and absolute counts (percentages)

^+^SA, South Africa; ^++^NYHA, New York Heart Association (I, No limitation of physical activity; II, Slight limitation of physical activity; III, Marked limitation of physical activity; and IV, Unable to carry out any physical activity without discomfort); **Pulse rate more than 100 bpm, Systolic blood pressure less than 100 mmHg and or tamponade requiring centesis.

## Table S2: Chest X-ray changes in the study population by serological HIV status

| **Feature** | **HIV positive** | **HIV negative** | **P** |
| --- | --- | --- | --- |
| Number of patients  Cardiomegaly   - Yes - No   Pericardial calcification   - Yes - No   Active PTB*   - Yes - No | 51  45 (62.5)  6 (30.0)  0 (0.0)  51 (56.0)  23 (63.9)  28 (50.0) | 41  27 (37.5)  14 (70.0)  1 (100.0)  40 (44.0)  13 (36.1)  28 (50.0) | 0.01  0.22  0.19 |

Values are absolute counts (percentages)

* PTB: Pulmonary tuberculosis

## Table S3: Electrocardiographic changes in study population by serological HIV status

| **Feature** | **HIV positive** | **HIV negative** | **P** |
| --- | --- | --- | --- |
| Number of patients  PR segment deviation   - Yes - No   ST segment elevation   - Yes - No   Micro voltage   - Yes - No   Electrical alternans   - Yes - No   Atrial fibrillation   - Yes - No | 33  5 (45.5)  28 (48.3)  9 (81.8)  24 (41.4)  6 (40.0)  27 (50.0)  3 (60.0)  30 (46.9)  4 (40.0)  9 (49.2) | 36  6 (54.5)  30 (51.7)  2 (18.2)  34 (58.6)  9 (60.0)  27 (50.0)  2 (40.0)  34 (53.1)  6 (60.0)  30 (50.8) | 0.86  0.01  0.49  0.31  0.31 |

Values are absolute counts (percentages)

## Table S4: Results of pericardial fluid analyses by serological HIV status

| **Feature** | **HIV positive** | **HIV negative** | **P** |
| --- | --- | --- | --- |
| Pericardiocentesis  Indication:   - Diagnostic - Therapeutic   Pericardial aspirate analyses:  Adenosine deaminase (n)   - > 40 IU/L - < 40 IU/L   Ziehl-Neelsen stain for acid-fast bacilli (n)   - Positive - Negative   TB culture (n)   - Positive - Negative | 18 (34.0)  8 (40.0)  10 (66.7)  5 (83.3)  5 (55.6)  3 (50.0)  15 (50.0)  4 (80.0)  3 (75.0) | 17 (39.5)  12 (60.0)  5 (33.3)  1 (16.7)  4 (44.4)  3 (50.0)  15 (50.0)  1 (20.0)  1 (25.0) | 0.57  0.12  0.18  0.67  0.72 |

Values are absolute counts (percentages)

IU/L: international units per litre.
